# Correspondence On: Effect of abdominoplasty with transverse plication on rectus diastasis

**DOI:** 10.1016/j.jpra.2026.03.012

**Published:** 2026-03-11

**Authors:** Francisco J. Villegas

**Affiliations:** Universidad del Valle, Cali, Colombia Tuluá, Valle del Cauca, Colombia

*Dear Sir,*

I read with great interest the article by Descarrega et al., “Effect of abdominoplasty with transverse plication on rectus diastasis,” published in *JPRAS Open*.^1^ The authors should be congratulated for addressing a controversial topic through a well-designed prospective clinical study complemented by a cadaveric model, providing robust insight into the true effect of transverse plication (TP) on inter-recti distance (IRD) during abdominoplasty.

TULUA lipoabdominoplasty, referenced in the article, is sometimes misinterpreted as isolated transverse plication rather than as a structured surgical philosophy representing a coordinated set of principles: horizontal infraumbilical plication, avoidance of supraumbilical undermining, unrestricted liposuction, neo-umbilicoplasty with independent determination of the new umbilical position, and low transverse abdominal scar placement, also determined by the surgeon rather than by flap tension after advancement. Within this approach, TP is performed as a broad, elliptical infraumbilical imbrication that shortens the elongated rectus muscles by recruiting the anterior rectus sheath and advancing the external oblique musculature, thereby achieving a multivectorial correction of abdominal wall laxity rather than a correction limited solely to rectus diastasis.^2^

The present study clearly demonstrates that TP produces a significant reduction in IRD in patients and cadaveric specimens, delineating its indications; however, the authors emphasize that vertical plication (VP), either alone or combined with TP, remains necessary when preoperative IRD exceeds 3 cm. This balanced conclusion brings welcome clarity to a field often marked by polarized positions. The authors also acknowledge that TP rarely reduces IRD below 1 cm, underscoring that TP does not aim to recreate a completely fused *linea alba* but rather to restore a functional abdominal wall.

It is also worth noting that the authors deliberately excluded liposuction from their clinical series, a methodological choice that strengthens the internal validity of their observations but may partially explain the transient epigastric redundancy reported in some patients. In our clinical experience, supported by objective measurements, we have consistently observed clear improvement in epigastric redundancy and effective avoidance of the midline “dome effect” or central fullness classically associated with isolated vertical plication and restricted or omitted liposuction in conventional abdominoplasty. We believe that this observation in TULUA cases is largely attributable to the unrestricted use of liposuction in the supraumbilical flap, which allows uniform flap thickness and improved flap redraping.

Rather than relying solely on a numerical threshold of 3 cm, we define pathological diastasis as a condition that is clinically evident on physical examination or identified intraoperatively by the presence of a visible midline bulge. In such cases, a combined TP and selective VP, performed through a limited dissection tunnel, has been required in approximately 10% of TULUA cases. This strategy allows correction of epigastric laxity while preserving the advantages of TP, including, no undermining above the umbilicus, reduced wound-closure tension and maintenance of vascular safety, representing an extension rather than a contradiction of the principles of transverse plication.^3^^,^^4^

In a consecutive series of 176 patients, we demonstrated that TP is associated with a significant reduction in waist circumference as well as a measurable decrease in the tension required for wound closure, dynamically assessed during surgery. These findings suggest that TP redistributes abdominal wall forces in a transverse and inferomedial direction, contributing not only to waist narrowing but also to a low, tension-free closure. In this context, unrestricted liposuction further enhances waistline reduction^5^ Figure 1.

**Figure 1:**
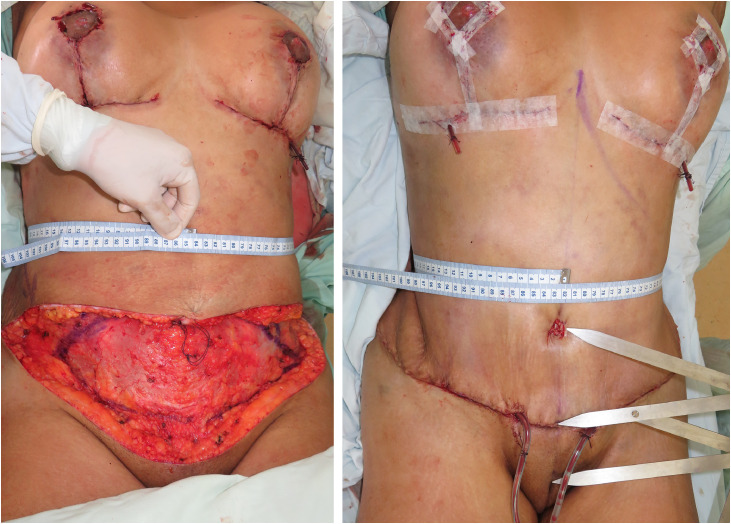
Intraoperative waistline measurements during TULUA abdominoplasty before and after transverse plication demonstrate a measurable reduction in waist circumference, attributable to inferomedial advancement of the external oblique muscles. On the right, a Fibonacci compass illustrates surgeon-controlled proportional positioning of the neo-umbilicus.

This reduction in wound-closure tension may confer additional benefits, including preserved flap perfusion, reduced dead space, and a potential decrease in wound- and scar-related complications such as dehiscence, scar widening, high scar placement, and upward traction of the genitalia. Together, these mechanisms may explain the favorable aesthetic outcomes and the low incidence of vascular complications observed in our series Figure 2.

**Figure 2:**
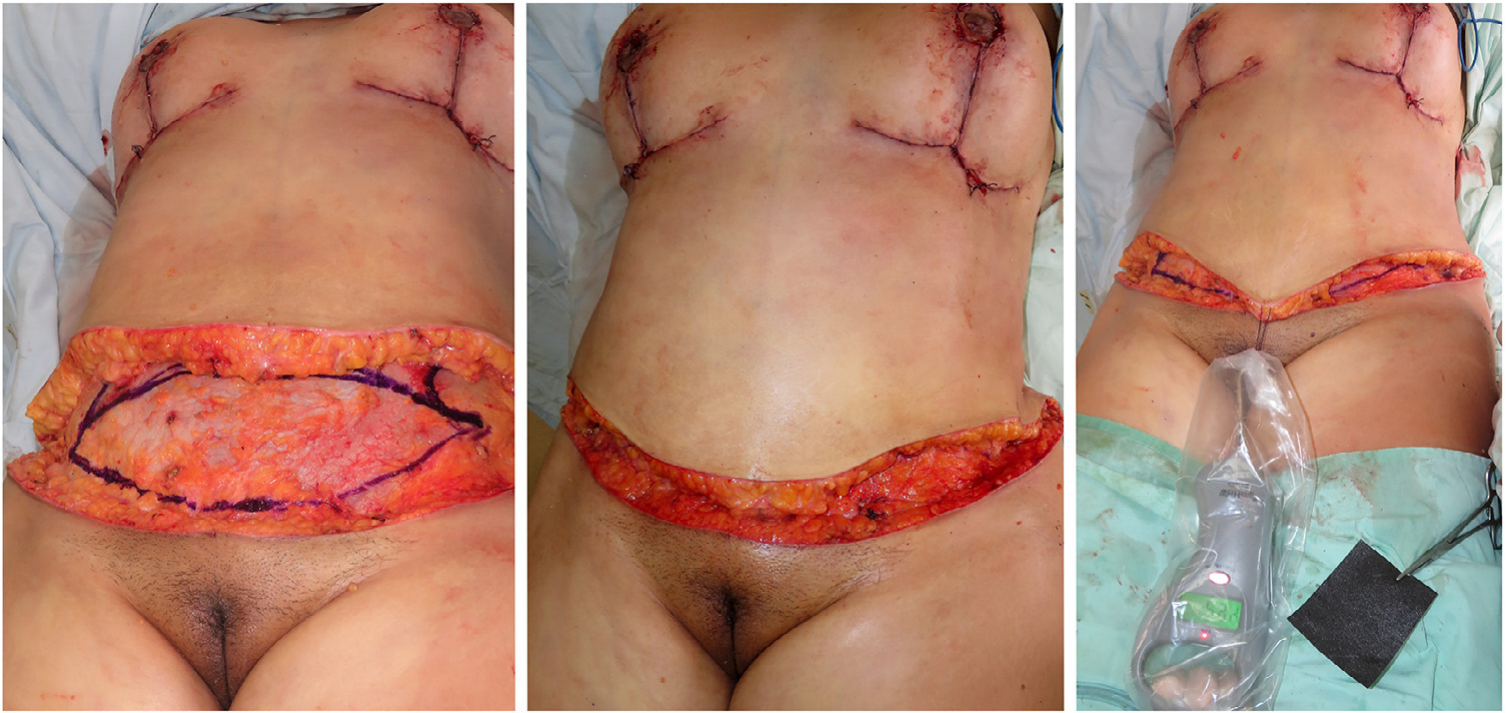
During TULUA abdominoplasty, transverse plication results in visible advancement of the supraumbilical flap with a corresponding decrease in wound-closure tension. Objective intraoperative dynamometric measurements, shown on the right, confirm this reduction.

A defining feature of TULUA is the avoidance of supraumbilical flap elevation, thereby preserving perforator vascularity. Although cadaveric studies remain uncommon in aesthetic and reconstructive abdominal wall surgery, they provide an essential bridge between theoretical concepts and clinical practice. This model opens promising avenues for future research, including comparative evaluation of flap perfusion with and without supraumbilical undermining, analysis of vector force redistribution, objective assessment of waistline reduction, potential effects on the inguinal canal region, and the interaction between transverse and vertical plication patterns.

By integrating a prospective clinical study with cadaveric validation, the authors establish a valuable reference model that is likely to stimulate further anatomical, biomechanical, and clinical research. They are to be congratulated for advancing the understanding of abdominal wall surgery and for opening new avenues of investigation grounded in both scientific rigor and surgical reality.

## AI disclosure statement

During the preparation of this work, the author used ChatGPT to improve language, grammar, and readability. Following the use of this tool, the author critically reviewed and edited the manuscript as required and assumes full responsibility for the content of the published article.

## Ethical approval

Not required.

## Declaration of competing interest

None declared
